# Hydration, Soundness, and Strength of Low Carbon LC^3^ Mortar Using Waste Brick Powder as a Source of Calcined Clay

**DOI:** 10.3390/ma18153697

**Published:** 2025-08-06

**Authors:** Saugat Humagain, Gaurab Shrestha, Mini K. Madhavan, Prabir Kumar Sarker

**Affiliations:** 1 School of Civil & Mechanical Engineering, Curtin University, Kent Street, Bentley, WA 6102, Australia; gaurab556@gmail.com (G.S.); p.sarker@curtin.edu.au (P.K.S.); 2 Department of Civil Engineering, Amrita School of Engineering, Amrita Vishwa Vidyapeetham, Coimbatore 641112, India; k_mini@cb.amrita.edu

**Keywords:** limestone calcined clay cement, waste brick powder, hydration, soundness, strength

## Abstract

The construction industry is responsible for 39% of global CO_2_ emissions related to energy use, with cement responsible for 5–8% of it. Limestone calcined clay cement (LC^3^), a ternary blended binder system, offers a low-carbon alternative by partially substituting clinker with calcined clay and limestone. This study investigated the use of waste clay brick powder (WBP), a waste material, as a source of calcined clay in LC^3^ formulations, addressing both environmental concerns and SCM scarcity. Two LC^3^ mixtures containing 15% limestone, 5% gypsum, and either 15% or 30% WBP, corresponding to clinker contents of 65% (LC^3^-65) or 50% (LC^3^-50), were evaluated against general purpose (GP) cement mortar. Tests included setting time, flowability, soundness, compressive and flexural strengths, drying shrinkage, isothermal calorimetry, and scanning electron microscopy (SEM). Isothermal calorimetry showed peak heat flow reductions of 26% and 49% for LC^3^-65 and LC^3^-50, respectively, indicating a slower reactivity of LC^3^. The initial and final setting times of the LC^3^ mixtures were 10–30 min and 30–60 min longer, respectively, due to the slower hydration kinetics caused by the reduced clinker content. Flowability increased in LC^3^-50, which is attributed to the lower clinker content and higher water availability. At 7 days, LC^3^-65 retained 98% of the control’s compressive strength, while LC^3^-50 showed a 47% reduction. At 28 days, the compressive strengths of mixtures LC^3^-65 and LC^3^-50 were 7% and 46% lower than the control, with flexural strength reductions being 8% and 40%, respectively. The porosity calculated from the SEM images was found to be 7%, 11%, and 15% in the control, LC^3^-65, and LC^3^-50, respectively. Thus, the reduction in strength is attributed to the slower reaction rate and increased porosity associated with the reduced clinker content in LC^3^ mixtures. However, the results indicate that the performance of LC^3^-65 was close to that of the control mix, supporting the viability of WBP as a low-carbon partial replacement of clinker in LC^3^.

## 1. Introduction

The construction industry is responsible for 39% of global carbon dioxide emissions, with concrete being the most widely used construction material [1]. Cement production alone accounts for 5 to 8% of global emissions [2]. As the principal binding component of concrete, ordinary Portland cement (OPC) contributes significantly to the environmental footprint of the built environment. Global strategies to mitigate this impact focus on reducing cement usage while maintaining satisfactory mechanical properties and the durability of the concrete. In this respect, the incorporation of supplementary cementitious materials (SCMs) as cement replacements has been studied extensively. However, the quantity of available good quality SCMs for the global cement replacement strategy is limited and constrained by geographic and industrial factors, necessitating the exploration of abundant and reactive alternatives [3,4,5]. Limestone calcinated clay cement (LC^3^) is a blended binder system developed to address both environmental and resource-related challenges. Limestone is globally more abundant than commercially available SCMs such as fly ash, slag, and silica fume [3]. However, using more than 10% limestone replacement causes soundness issues in concrete and increased porosity. To mitigate this disadvantage, calcined clay is incorporated into LC^3^ concrete [6]. The advantages of using LC^3^ binders include their abundant availability, sustainability as a low-carbon cement option, and ease of production [7].

LC^3^ is a ternary blended binder comprising clinker, calcined clay (mainly metakaolin), and limestone, with added gypsum. It offers up to a 40% reduction in CO_2_ emissions compared to OPC, without adversely affecting strength or durability [8,9]. The synergy between limestone and calcinated clay enhances early hydration through the formation of carbo-aluminate phases (carbonates from limestones react with alumina from calcinated clay), which densifies the microstructure and contributes to the mechanical strength and durability of concrete [8,10,11]. The performance of LC^3^ binder incorporating commercial SCMs and other curing conditions has been investigated in recent studies. Guo et al. [12] showed that the inclusion of slag in LC^3^ improved ettringite stability and enhanced long-term compressive strength. Recent studies have also evaluated the volumetric stability and shrinkage behavior of LC^3^-based systems. Wyrzykowski et al. [13] reported that LC^3^ concretes exhibited higher plastic shrinkage compared to OPC and Portland–limestone cement (PLC), attributed to slower early-age stiffness gain and finer cement particles. Liang et al. [14] investigated strain-hardening engineered cementitious composites (ECCs) based on LC^3^ and observed that mixtures with an adjusted LC^3^ and fly ash ratio displayed improved tensile strain capacity, highlighting the importance of microstructural control.

Most LC^3^ studies rely on metakaolin as the calcined clay source. However, the production of metakaolin involves the high-temperature treatment of kaolin, leading to elevated energy demand and associated CO_2_ emissions. Additionally, the limited geographic availability and high cost of metakaolin restrict its large-scale use in low-income and developing areas [8,15]. Meanwhile, a large quantity of waste brick material is generated globally from the demolition of old brick structures, natural disasters, and construction works, especially in regions with older clay-based masonry infrastructures [16]. Waste brick powder (WBP), when properly processed, offers pozzolanic reactivity due to the presence of aluminosilicate phases formed during brick firing. Unlike metakaolin, WBP is already calcined during brick manufacturing and can be repurposed with minimal processing, making it a low-cost, energy-efficient, and sustainable alternative [17]. Some researchers have utilized WBP as a partial cement or fine aggregate replacement in conventional concrete [18,19]. Recent studies have further highlighted the potential of WBP in low-carbon binder systems, contributing to circular economy frameworks and reducing raw material use and emissions [20,21]. However, its incorporation in LC^3^ formulations remains underexplored. While Wang et al. [22] reported moderate hydration activity of finely ground brick powder under ambient curing, there remains a lack of comprehensive studies evaluating its performance in LC^3^ formulations across different clinker replacement levels, particularly with respect to hydration kinetics, mechanical strength development, and porosity evolution while using WBP instead of commercial metakaolin. Moreover, most prior LC^3^ studies have used commercial metakaolin and standardized limestone sources, often overlooking the variability inherent in waste-derived materials. There is also limited insight into how brick powder influences the hydration kinetics, strength development, and porosity when used as the primary alumina source in LC^3^ binders. This clearly defines a knowledge gap in terms of understanding the feasibility and performance of WBP-derived calcined clay in LC^3^ systems. This gap is especially critical considering its use in structural concrete. In addition to mechanical performance, the environmental and practical implications of clinker substitution are significant. The clinker substitution strategy adopted in the LC^3^-65 mix, which incorporated 15% limestone and 15% waste brick powder (WBP), is estimated to result in a 25–30% reduction in CO_2_ emissions relative to ordinary Portland cement, as supported by earlier studies on LC^3^ systems [2,8]. Valorizing WBP utilizes demolition waste from landfills, reduces landfill burden, and reduces embodied energy, enabling cost-effective low-carbon construction in resource-limited contexts. By exploring the use of WBP in LC^3^, this study aims to contribute to resource-efficient construction practices, as this material may be a suitable alternative for masonry, mortar beds, and low-load structural components. The pilot-scale manufacture of LC^3^ cements in India has further validated their feasibility for low-rise housing and infrastructure projects under real-world construction conditions [7].

In this context, the present study investigated the feasibility of using WBP as a calcined clay source in LC^3^ systems. The hydration and mechanical characteristics of two LC^3^ binders were evaluated in comparison to a general purpose (GP) cement mortar used as the control. Thus, this study addresses the research gap by investigating the performance of waste brick powder as a source of calcined clay in LC^3^ mixtures and thereby contributing to developing a low-cost, low-carbon cementitious binder that can support sustainable development in regions with limited access to commercial SCMs. The outcomes of this study have practical implications for circular economy practices and carbon footprint reduction in the construction sector.

## 2. Materials and Methods

### 2.1. Materials

The general purpose (GP) cement used in this study was bought from local suppliers that comply with standard AS 3972 [23] to make a control mix. The clinker was obtained from the local cement company that manufactures GP cement. Waste bricks were collected from a local demolition and salvage company. The presence of Al_2_O_3_ in WBP is expected to support pozzolanic interactions when used in LC^3^ systems [20]. Waste bricks were ground to powder using a ball mill. Limestone was acquired from a local supplier that meets the Australian standard AS 1672.1 [24]. Natural sand with a fineness modulus (FM) of 2.89 and complying with Australian standard AS 2758 [25] was used as the filler in the mortar mixtures. The chemical compositions of the clinker, limestone, gypsum, and GP cement are shown in Table 1.

### 2.2. Preparation of LC^3^ Binder and the Mix Proportions

**Figure 1:**
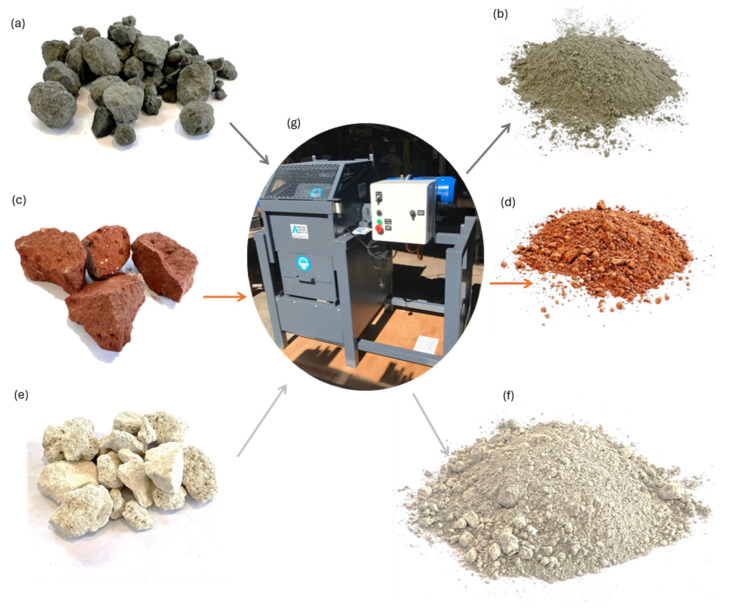
Visual representation of the mechanical grinding process used for raw binder components. (**a**) Clinker, (**c**) waste brick, and (**e**) limestone were milled using (**g**) laboratory-scale ball mill to obtain finely ground powders, (**b**) clinker powder, (**d**) WBP, and (**f**) limestone powder.

All the raw materials used for making the LC^3^ binders (clinker, waste brick fines, limestone, and gypsum) were nodular and had a non-uniform size distribution. These raw materials were crushed separately using a 50 L batch ball mill (BBM50) to produce powders with a uniform size distribution, as shown in Figure 1. Each material was ground at a rotational speed of 60 rpm using a material-to-charge ratio of 1:5. The grinding media (charge) comprised stainless steel balls, with 60% of the total weight consisting of 50 mm diameter balls and the remaining 40% composed of 25 mm diameter balls. Each raw material was then precisely weighed and thoroughly mixed to obtain a consistent binder mix in various proportions, as shown in Table 2. Subsequently, the binder was mixed with sand at a ratio of 1:3, which is commonly used for small to medium construction work, particularly in bricklaying and plastering. This study adopted a water-to-binder ratio of 0.5, as previous research indicated that a slightly higher water-to-binder ratio was used in the LC^3^ mortar system compared to the GP system [26]. The mortar was mixed in a Hobart mixer and cast, compacted, demolded, and cured as per AS 1012.8.3 [27]. Mortar specimens were cast in three different mold sizes based on the intended test: 50 mm × 50 mm × 50 mm cubes were used for compressive strength testing, 25 mm × 25 mm × 285 mm prisms were used for drying shrinkage tests, and 40 mm × 40 mm × 160 mm prisms were used for flexural strength tests. The samples were allowed to set for 24 h before being demolded and submerged under water at room temperature until the day of testing. The control, LC^3^-65, and LC^3^-50 mortar samples were prepared and subjected to various tests, as detailed in Section 2.3.

**Table 1:** Chemical compositions of materials.

Ingredient	C_3_S	C_2_S	C_4_AF	CaO	SiO_2_	CaCO_3_	Al_2_O_3_	Fe_2_O_3_	CaSO_4_
Clinker	67.5	16	8.5	2	0.5	-	-	-	-
Limestone	-	-	-	-	1.5	95	0.75	0.5	-
Gypsum	-	-	-	-	1.5	-	-	-	97
Cement	-	-	-	63.1	20.7	-	5.70	2.90	-
WBP [28]	-	-	-	2.06	66.21	-	18.86	6.4	-

**Table 2:** Mass proportions (%) of materials in LC^3^ binders.

Binder Mix ID	Clinker	Waste Brick Powder	Limestone	Gypsum
LC^3^-50	50	30	15	5
LC^3^-65	65	15	15	5

### 2.3. Test Methods

**Figure 2:**
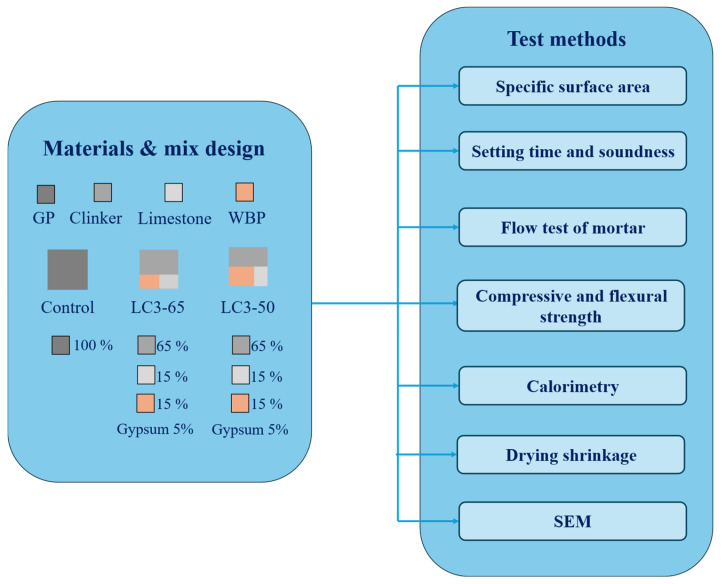
Mix compositions and corresponding test methods used in the experimental program.

A schematic overview of the mix design and associated test methods is provided in Figure 2. The procedures include standard tests for physical and mechanical properties, calorimetry, and SEM analysis. Details are given in the following subsections.

#### 2.3.1. Blaine’s Fineness Test

The Blaine’s fineness test was conducted to determine the specific surface area of the binders using a CL 14601 air permeability apparatus (CiviLab, Mount Kuring-gai, Australia) in accordance with AS 2350.8 [29]. It is important to know the specific surface area of LC^3^ binders as it affects the water demand in mortar [30]. This test is commonly referred to as the air permeability test, which measures the time it takes for the pressure drop between two standard values after a known volume of air passes through the compacted powder held by a cell [31]. In the procedure, a circular piece of filter paper provided with the apparatus was placed at the bottom of the sample cell. The binder was then inserted into the cell, and a plunger was used to compress the powder bed. The sample cell was subsequently positioned on the apparatus and secured firmly. The manometer fluid was raised to the start mark using the inflation bulb. After that, the valve was opened to allow air to pass through the sample cell, which caused the manometer fluid to drop. The time difference between the moments when the lower meniscus of the manometer fluid reached levels A and B was recorded. This test was repeated three times for each sample. The specific surface area was determined by using Equation (1).

(1) $$S=\frac{K}{\rho }\frac{\sqrt{e^{3}}}{\left(1-e\right)}\frac{\sqrt{t}}{\sqrt{10*\eta }}$$

where

S = specific surface area in m^2^/kg, K = apparatus constant = 1268.31, *ρ* = density of cement, e = porosity (assumed to be 0.5), t = time difference, η = air viscosity (assumed to be 0.0000157 m^2^/s).

#### 2.3.2. Setting Time and Soundness Test of Binder Paste

**Figure 3:**
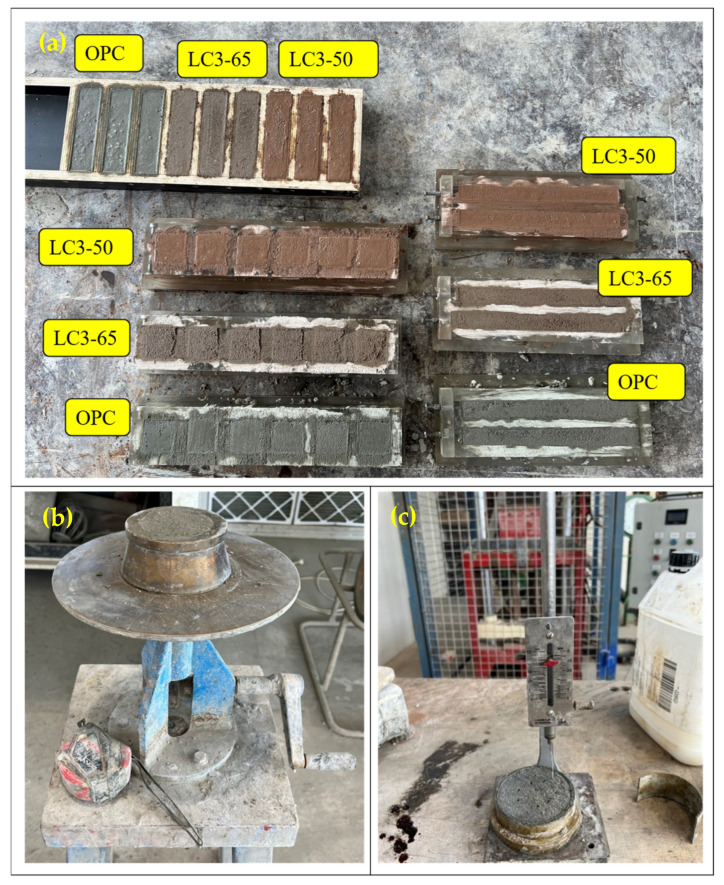
Experimental method: (**a**) Mortar samples for compressive, flexural, and drying shrinkage tests; (**b**) flow test of mortar; (**c**) setting time test.

The setting time of the binder paste was measured using a Vicat’s apparatus (Figure 3) as per AS/NZS 2350.4 [32]. Knowledge of the setting time is necessary for scheduling stages of concrete construction [33]. The initial setting time was determined by measuring the duration for the paste to stiffen enough to resist 5 mm penetration. The final setting time was recorded when the paste stiffened sufficiently to support the needle mass without penetration. The soundness test was performed in mortar samples. Excess lime in the binder can lead to the expansion of paste upon setting, potentially causing cracks that affect both the mechanical and durability aspects of the structure. Therefore, a binder that contains no extra lime is considered sound. To evaluate this, a soundness test was carried out using the Le Chateliers apparatus, following the guidelines of AS 2350.5 [34], wherein paste-filled molds were immersed in water for 24 h and subsequently boiled for 3 h. The measured expansion of the indicator arms reflected the volumetric change of the binder. Minimal expansion confirmed the absence of free lime and satisfactory soundness.

#### 2.3.3. Flow Test of Mortar

The flow table test was conducted to evaluate the workability of the mortar mixture complying with Australian standard 2701 [35]. A higher flow indicates more workability. To perform this test, the freshly prepared mortar was filled in the mold and compacted using a tamping rod. Excess mortar was then scraped off from the top by rotating and lifting it upward. Then, the flow table apparatus was dropped 25 times in 15 s, and the spread diameter of the mortar was measured along two orthogonal directions.

#### 2.3.4. Compressive and Flexure Strength Tests

The compressive strength of the mortar was measured at both 7 days and 28 days using the Shimadzu 300 kN UTM machine (Kyoto, Japan) (Figure 3), following AS 1012.9 [36]. The loading rate during the test was 20 MPa per minute. A flexure test was conducted on a sample measuring 40 mm × 40 mm × 160 mm at 28 days using the same machine complying with modified AS 1012. 11 [37] and Atis et al. [38].

#### 2.3.5. Calorimetry Test

In this study, a TAM Air TA calorimeter (TA Instruments, New Castle, DE, USA) was used to measure the heat of hydration of the binder in accordance with the ASTM C1679 standard [39]. This was performed to gain insights into the hydration process by analyzing the rate of heat produced and the cumulative heat released from the binder materials during the hydration. All three binder pastes were prepared with a water-to-binder ratio of 0.5 and were thoroughly mixed with a glass rod to achieve a uniform consistency. A 6 gm sample of homogenous paste was placed into the sample ampule, sealed, and positioned in the calorimeter. The rate of heat generation and the cumulative heat of hydration were studied for 72 h, following a procedure similar to that in previous studies [28].

#### 2.3.6. Drying Shrinkage Test

Drying shrinkage in concrete occurs due to the loss of adsorbed water from hydrated products. Understanding the shrinkage behavior of LC^3^ mortar is crucial because shrinkage often leads to cracking, which can result in increased durability issues [40]. This study measured shrinkage in the 25 mm × 25 mm × 285 mm cement mortar with shrinkage gauge studs placed at both ends during casting. The samples were demolded after 24 h and then immersed in water for 7 days to ensure sufficient early hydration. Following this period, the samples were exposed to ambient conditions of 23 ± 2 °C and 50 ± 5% relative humidity, and length change measurements were recorded at regular intervals (1, 3, 7, 14, 28, and 56 days) using a vertical comparator digital gauge in accordance with AS 1012.13 [41].

#### 2.3.7. Scanning Electron Microscopy (SEM)

SEM of samples was conducted using a TESCAN VEGA3 instrument (Brno, Czech Republic) to investigate the mortar microstructures and correlate them with the mechanical and chemical properties. To ensure conductivity, the samples were coated with carbon. The electron beam was operated at an accelerating voltage of 15 kV for both secondary and backscattered electron modes. The SEM images were analyzed with TBitmap software to examine the porosity [42,43].

## 3. Results and Discussion

### 3.1. Specific Surface Area of Binders

The specific surface area results from the Blaines fineness test for GP cement, LC^3^-65 binder, and LC^3^-50 binder were 410, 371, and 389 m^2^/kg, respectively. This indicates that the cement particles were slightly finer than those of the LC^3^-65 and LC^3^-50 binders, providing a larger surface area. Additionally, the LC^3^-65 binder was finer than the LC^3^-50 binder. This is attributed to LC^3^-50 having a lower percentage of clinker than LC^3^-65, which increases the number of coarse materials and decreases the specific surface area. It is noted that all materials were ground in identical milling conditions for a fixed duration of 3 h; therefore, the variation in fineness is primarily influenced by the inherent differences in material composition rather than the grinding time.

### 3.2. Setting Time and Soundness

The initial setting time for the control paste was 150 min. In comparison, the mortars mixed with LC^3^-65 and LC^3^-50 had setting times of 165 and 180 min, respectively. The final setting times were recorded at 210 for control, 240 min for binder with LC^3^-65, and 270 min for binder with LC^3^-50. The quicker setting time of GP cement can be attributed to the fine materials, which provide a larger surface area for water to interact with. This accelerates the hydration process and results in a faster setting. Notably, the binder material with 30% brick powder set more slowly than the binder with 15% brick powder. This difference is likely due to the slower hydration rates of brick powder compared to clinkers, resulting in a faster hydration rate in binders with a higher clinker content. Sharma et al. [9] also reported similar observations in LC^3^ systems, where increasing the calcined clay content delayed the setting due to slower aluminate reactivity. The expansion in Le Chateliers test is limited to 10 mm [44]. The expansion measured was 1.5 mm for GP cement, 1 mm for LC^3^-65 binder, and 0.5 mm for the LC^3^-50 binder, indicating that all the values were well below the 10 mm limit. Additionally, the soundness of LC^3^-50 was lower than that of LC^3^-65, suggesting a reduced lime content. This implies that less lime is consumed with clay to form carbo-aluminates [3].

### 3.3. Workability of Mortars

The flow diameters for the GP, LC^3^-65, and LC^3^-50 mortar mixes were 53.5, 53.5, and 59.5 mm. The comparable values for GP and LC^3^-65 indicate a minimal impact of 15% WBP replacement on flowability. In contrast, the 10% increase in flow diameter in LC^3^-50 can be attributed to the reduced clinker content. Since clinkers react faster with water, reducing the clinker content leads to a decreased water demand, which allows for more water in the mix and consequently increases the flow rate.

### 3.4. Compressive and Flexure Strengths

**Figure 4:**
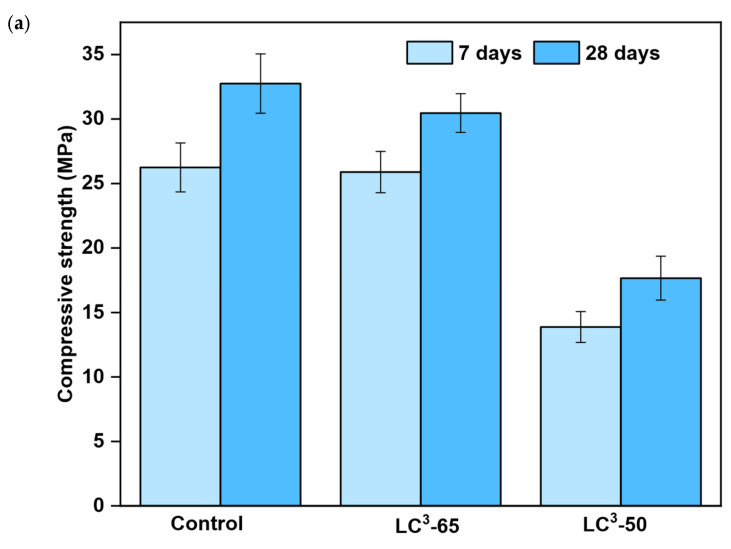

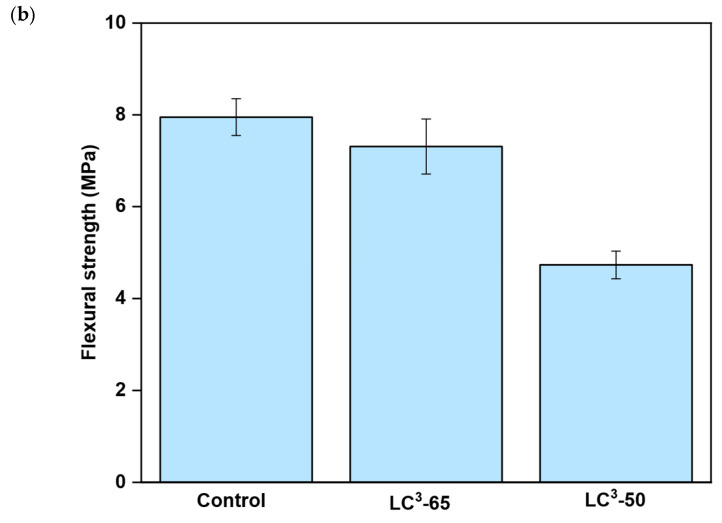
(**a**) Comparison of 7-day and 28-day compressive strengths; (**b**) comparison of 28-day flexural strengths.

The 7- and 28-day compressive strengths of the mortar samples are plotted in Figure 4. At 7 days, the control, LC^3^-65, and LC^3^-50 mixes achieved compressive strengths of 26.2 MPa, 25.8 MPa, and 13.9 MPa, respectively. At 28 days, the strengths were 32.7 MPa for the control, 30.4 MPa for LC^3^-65, and 17.6 MPa for LC^3^-50. At 7 days, the LC^3^-65 and LC^3^-50 mixes showed 1.5% and 47% lower compressive strengths than the control mix; at 28 days, the reduction was 7% and 46%. The strength gains in the mixes from 7 days to 28 days were 25%, 18%, and 27%, respectively. The control mix continued to gain strength up to 28 days, while the strength gain in the LC^3^-65 mix was observed to be lower. Although all mixes gained strength, LC^3^-65 had a slower gain than the control. LC^3^-50 exhibited a similar gain despite its lower strength value, indicating slower hydration due to the high clinker substitution with calcined clay. In line with this trend, Dhandapani and Santhanam [45] reported a 31% lower 28-day compressive strength when 30% calcined clay was used in LC^3^ mortars compared to the control, reinforcing the observation that high levels of clinker replacement result in strength reduction. However, in a separate study by Dhandapani et al. [46], the LC^3^-50 binder had a higher strength than OPC, due to the optimized sulfate aluminate ratio. Shree et al. [47], using metakaolin in LC^3^ mixtures, reported a 28-day compressive strength that was 12% higher than their OPC control mix at 50% clinker replacement, whereas LC3-50 in this study showed a 46% reduction. This contrast emphasizes the critical role of the kaolinite content and reactivity, with WBP exhibiting significantly lower reactivity than metakaolin. This performance drop may also be attributed to sulfate imbalance. As Scrivener et al. [8] reported, adequate sulfate is essential to control the reaction of reactive aluminates from calcined clays. An improper balance can lead to premature monosulfate formation or delayed ettringite formation, both of which hinder the development of stable carboaluminates and reduce strength gain.

The 28-day flexural strength test results, shown in Figure 4, revealed that the control mix had the highest flexural strength of 8 MPa. The flexural strength of the LC^3^-65 mix was 8% less than that of the control mix, while the LC^3^-50 mix showed a reduction of 40.5% compared to the control mix. These findings indicate that while LC^3^-65 maintains structural integrity, the LC^3^-50 mix suffers from insufficient binder reactivity due to higher clinker substitution. These results highlight the influence of the calcined clay-to-limestone ratio and clinker content on the mechanical performance. In the research by Nguyen et al. [48], a 2:1 ratio of calcinated clay to limestone was used, replacing up to 30% of cement. This mixture demonstrated similar strength to the control mix, indicating that a 2:1 ratio is ideal for the replacement. However, in this study, clinkers and LC^3^ mortars entirely replaced GP cement, altering the hydration reaction; therefore, the benefits observed with the 2:1 ratio of calcinated clay and limestone were not found. This disparity may also arise from differences in calcined clay reactivity; Nguyen et al. [48] used refined metakaolin, whereas this study utilized waste brick powder, which may possess lower pozzolanic activity. A study by Dixit et al. [49] conducted over 91 days showed a continuous increase in compressive strength in the control mix, while the strength gain for the LC^3^ mortar was minimal after 56 days. These compressive and flexural strength results provide insights into the ideal ratios of limestone and calcinated clay, alumina reactivity, and the appropriate replacement percentage of clinker in the mix to achieve better mechanical strength. They also highlight the need for long-term studies on the mechanical strength development of mixes made with LC^3^ binder and clinker compared to the control mix to further understand their long-term strength gain.

### 3.5. Calorimetry

**Figure 5:**
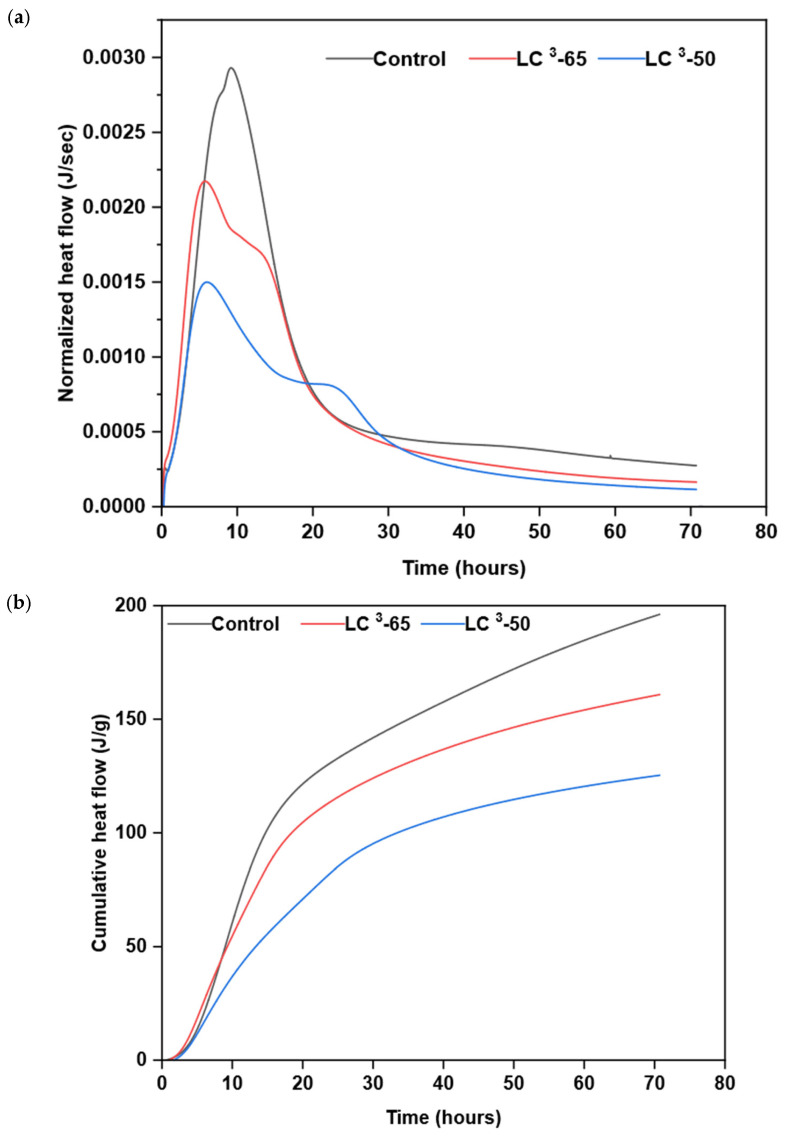
(**a**) Normalized heat flow and (**b**) cumulative heat flow of the binder paste.

The peak heat flow rate and the cumulative heat flow obtained from the calorimetry result are shown in Figure 5. The control mix has the highest peak heat of hydration rate, measured at approximately 0.003 J/s. In comparison, mixtures LC^3^-65 and LC^3^-50 had 33% and 50% lower peak heat flow rates, respectively. The time taken to reach the peak heat flow rate for the control mix was approximately 9.5 h, whereas for the LC^3^-65 and LC^3^-50 mixes, the time was around 6 h, indicating accelerated early hydration in LC^3^ systems. The cumulative heat of hydration for the control mix was 190 J/gm, while LC^3^-65 and LC^3^-50 exhibited 15% and 34% decreases, respectively. These results are consistent with previous findings, such as Avet et al. [50], reporting that LC^3^ systems exhibit lower total heat release than OPC due to the dilution of clinker and the slower pozzolanic reactions of calcined clay. Unlike the control mix, which exhibited a sharp decline after the peak, LC^3^-65 and LC^3^-50 demonstrated a more gradual post-peak heat flow curve, indicating a slower consumption of hydration products and extended reaction kinetics, due to the presence of brick powder and limestone compared to clinker. This effect was more pronounced in the LC^3^-50 mix, as it contains a higher percentage of brick powder than the LC^3^-65 mix. Additionally, the peak rate of hydration was lowest in LC^3^-50 due to the lower percentage of clinkers. The heat flow rate for LC^3^-50 was 31% less than LC^3^-65 when the clinker amount was 15% less. Although the LC^3^ mixes exhibited lower peak heat flow, the peak occurred earlier. This is likely due to the early consumption of clinkers due to hydration. The high peak hydration rate observed in the control mix, followed by LC^3^-65 and LC^3^-50, also explains the compressive strength obtained in this test. The compressive strength of the control mix was higher than that of the other two mixes due to rapid hydration, with LC^3^-65 and LC^3^-50 following. Notably, the early compressive strength of the LC^3^-50 mix was 47% lower, as discussed in the previous section, corresponding with the heat flow rate, which was approximately 50% lower. Additionally, the cumulative heat flow of the control mix was higher than that of the other mix, indicating a higher degree of hydration. This can be attributed to the slower reactivity of clinker compared to cement, which is influenced by particle size, and the reaction involving limestone and calcinated clay is slower than the hydration reaction. The slope of the cumulative heat flow is still higher in the control mix, demonstrating a longer and sustained hydration process, which is linked with the increase in compressive strength from 7 to 28 days.

### 3.6. Drying Shrinkage

**Figure 6:**
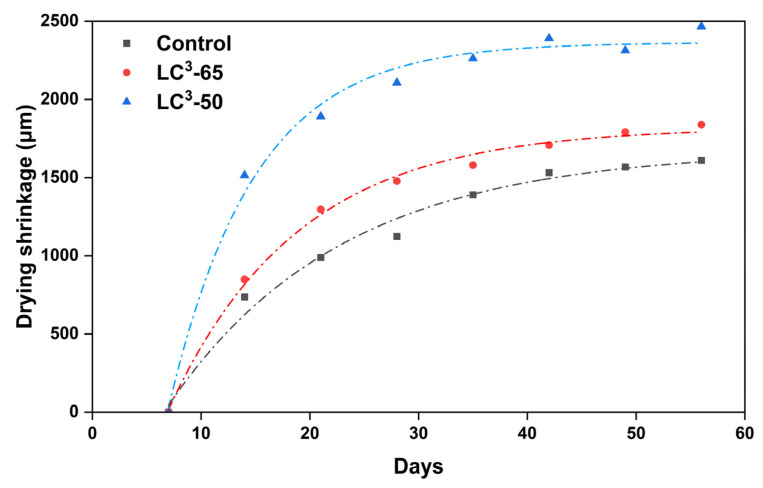
Drying shrinkage of OPC, LC^3^-65, and LC^3^-50 mortars.

The 56-day drying shrinkage values of all the mortar mixes are shown in Figure 6. The total shrinkage of the control mix was 1610 micro strains at 56 days. The LC^3^-65 mix exhibited a 14% increase, while the LC^3^-50 mix showed a 53% increase in total shrinkage compared to the control mix. The highest shrinkage was observed in the LC^3^-50 mix. The LC^3^-50 mix contains 50% clinker and 30% brick powder. The brick powder reacts slowly, leaving excess water in the mortar matrix. This results in the pore structure releasing more water during drying than the control mix, which enhances capillary tension, increases meniscus curvature, and results in greater shrinkage. Furthermore, the lower stiffness of the LC^3^-50 matrix at early ages may have contributed to increased deformability under drying stress, as the paste lacked sufficient early C-S-H formation. The reduced packing density due to the irregular particle shape and size of brick powder may also have increased the pore connectivity, amplifying water migration during drying. The progressive increase in shrinkage observed in both LC^3^ mixes correlates with their reduced cumulative heat release, as previously discussed. These findings are consistent with observations by Nguyen et al. [51] and Juenger et al. [52], where increased limestone or low-reactivity SCMs were shown to delay pore refinement and increase drying shrinkage susceptibility. Further, Brooks and Johari [53] reported that adding up to 15% metakaolin decreases the drying shrinkage. In contrast, other studies have shown that replacing cement with up to 10% limestone results in comparable shrinkage to control, but higher replacement levels tend to increase shrinkage [54,55]. Therefore, the combination of limestone and metakaolin can yield drying shrinkage values similar to or slightly lower than control systems [46]. However, in this study, WBP was used instead of metakaolin, which likely contributed to the higher shrinkage observed, due to its lower reactivity and irregular particle morphology, which affected the packing density and delayed hydration development.

### 3.7. Microstructure

**Figure 7:**
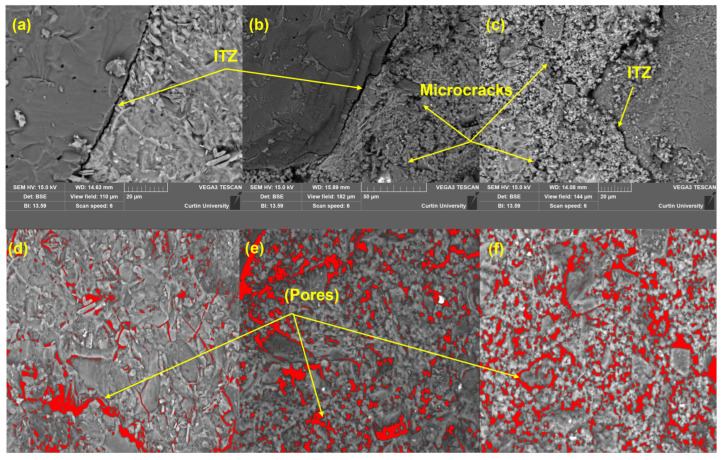
SEM image showing ITZ, microcracks, and pores. (**a**,**d**) Control, (**b**,**e**), LC^3^-65, (**c**,**f**) LC^3^-50.

The SEM images of the mortar samples are shown in Figure 7a–c. In the control mix, the ITZ exhibited fewer microcracks. In contrast, the LC^3^ binder samples displayed wide ITZ, with more interconnected microcracks within the ITZ. This suggests increased internal stress concentration due to non-uniform particle packing and delayed pozzolanic reaction, particularly in brick powder systems. The wider ITZ and the higher connections of microcracks support the compressive strength results obtained in this study. Using TBitmap software, the porosity was calculated to be 7% in the control sample, 11% in LC^3^-65, and 15% in the LC^3^-50 sample (Figure 7d–f). The increased pore volume in LC^3^ mixtures is attributed to incomplete clinker hydration and a coarser pore structure, consistent with the observed cumulative heat flow and shrinkage trends. Such microstructural characteristics are indicative of a lower packing density and reduced formation of C–S–H gel.

The higher porosity and wider ITZ observed in LC^3^-50 directly contributed to its reduced compressive strength and higher shrinkage, as the interconnected pores and microcracks facilitated greater moisture loss and reduced early stiffness. In contrast, the relatively denser matrix in LC^3^-65 helped retain strength closer to the control mix. This mechanistic relationship between the microstructural features and macroscopic performance highlights the influence of WBP incorporation on the hydration progression, matrix densification, and ultimately, the strength and shrinkage behavior of LC^3^ mortars.

## 4. Conclusions

This study investigated the use of 15% and 30% waste brick powder as the source of calcined clay on the properties of two LC^3^ mixtures containing 15% limestone and 5% gypsum, in comparison with a control mix. The key findings of this study are as follows:

- The compressive strength results showed that at 7 days, mortars LC^3^-65 and LC^3^-50 were 1.5% and 47% weaker than the control mix, respectively. The 28-day compressive strengths of the LC^3^ mixtures were 7% and 46% lower than those of the control mix. Similarly, the 28-day flexural strengths were 8% and 40% lower.
- The isothermal calorimetry results showed 33% and 50% lower peak heat flow rates for LC^3^-65 and LC^3^-50 compared to the control paste. Additionally, the cumulative heat flow values of the LC^3^ mixes were 15% and 34% less than the control, respectively. This result supports the lower strengths of LC^3^-50 and LC^3^-65 due to the slower hydration caused by increasing clinker replacement.
- Both LC^3^ mixtures passed the soundness test showing minimal expansion in the Le Chatelier apparatus. The 56-day drying shrinkage of the control mortar was 1610 micro strains. In contrast, mortars LC^3^-65 and LC^3^-50 exhibited 14% and 53% increases in drying shrinkage compared to the control, respectively.
- The SEM analysis revealed fewer microcracks and lower porosity in the control sample compared to the LC^3^ samples. The porosity values of the control mix, LC^3^-65, and LC^3^-50 were found to be 7%, 11%, and 15%, respectively.
- The reduction in strength in the LC^3^ mixtures is attributed to their slower hydration rate and increased porosity compared to the control. Nevertheless, the results indicate that the performance of LC^3^-65 was close to that of the control mix, supporting the potential use of WBP as a low-carbon partial replacement of clinker in LC^3^.

## Data Availability

The original contributions presented in this study are included in the article. Further inquiries can be directed to the corresponding author.
